# Psoriatic Onycho-Pachydermo Periostitis (POPP)

**DOI:** 10.31138/mjr.310724.iia

**Published:** 2025-05-14

**Authors:** Aline Serfaty, Flavia Costa, Luiza Feres, Alessandro Severo, Edson Marchiori, Clarissa Canella

**Affiliations:** 1 Universidade Federal do Rio de Janeiro, Rio de Janeiro, Brazil,; 2 Medscanlagos Radiology, Cabo Frio, Rio de Janeiro, Brazil,; 3 Diagnostico das Americas (DASa), Rio de Janeiro, Brazil,; 4 Clínica Dermatológica dra Vanessa Metz, Rio de Janeiro, Brazil,; 5 Universidade Federal Fluminense, Rio de Janeiro, Brazil

**Keywords:** POPP, psoriatic arthritis, onychodystrophy, ultrasonography, MRI

## CASE DESCRIPTION

**Figure 1.**
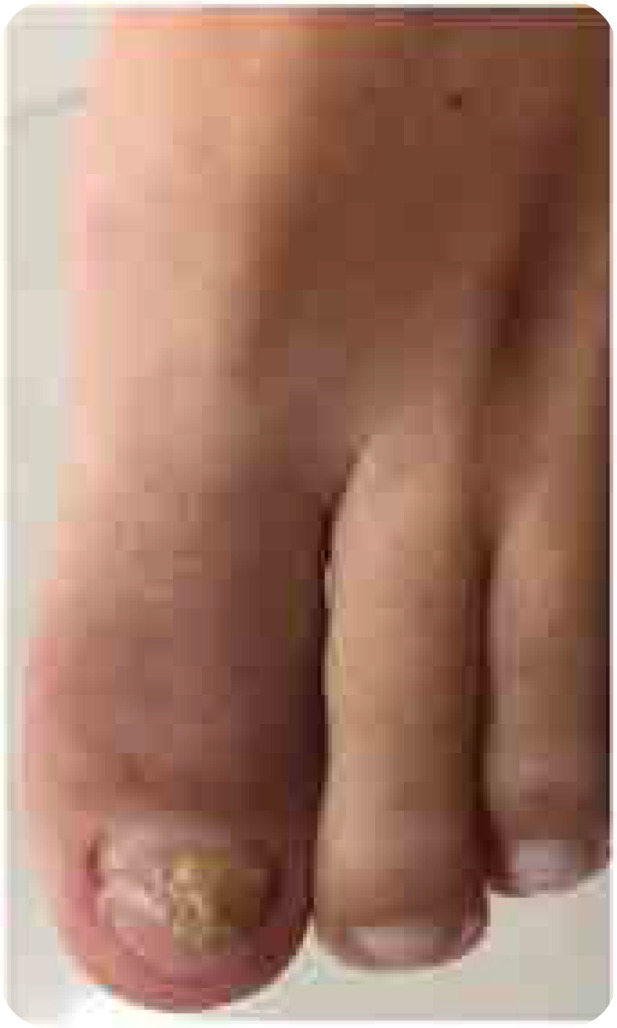
**(left).** Photograph of the patient’s left forefoot showing nail dystrophy, oedema, and erythema of the interphalangeal joint of the hallux.

**Figure 2.**
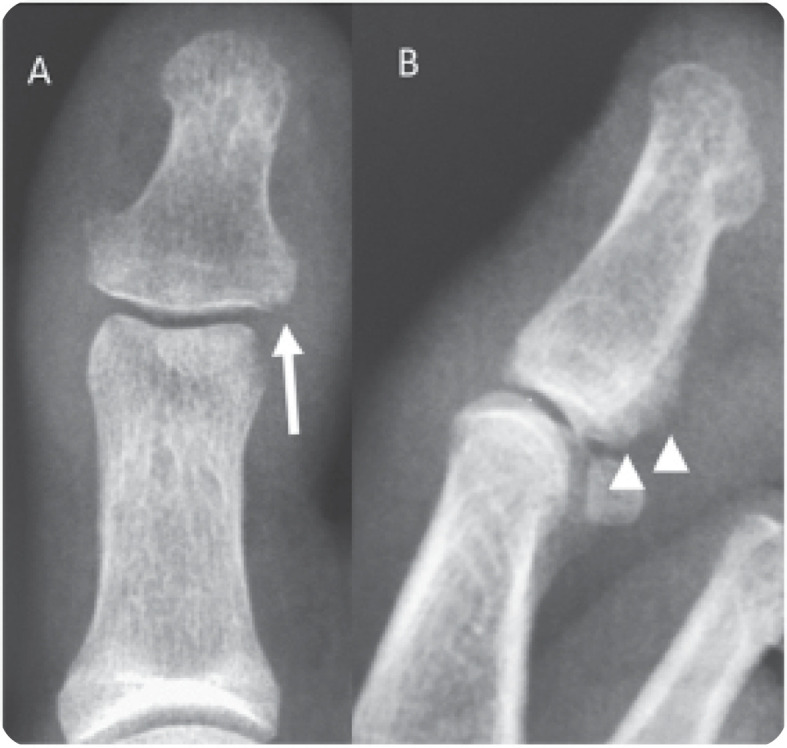
**(right).** Conventional radiography of the hallux with anteroposterior (A) and lateral (B) views showing periarticular erosions (arrows in A) and periosteal reaction (arrowheads in B) of the distal phalanx.

**Figure 3.**
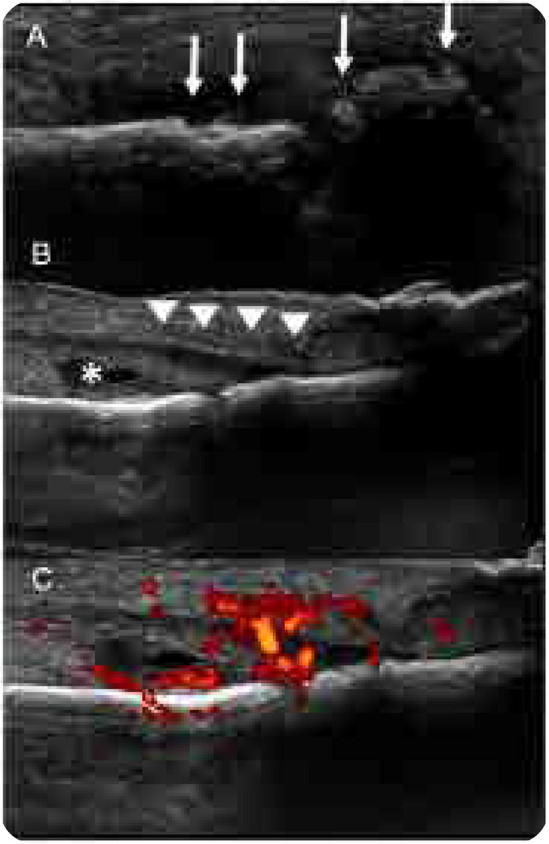
**(left).** Ultrasonography of the hallux revealing enthesitis characterised by hyperostosis of the proximal and distal phalanges (arrows in **A**), and increased thickness and hypoechogenicity of the extensor hallucis longus (arrowheads in **B**) associated with peritendinous positive Doppler findings adjacent to the tendon’s insertion at the base of the distal phalanx (**C**). Synovitis of the interphalangeal joint of the hallux (asterisk in **B**) is also visible.

**Figure 4.**
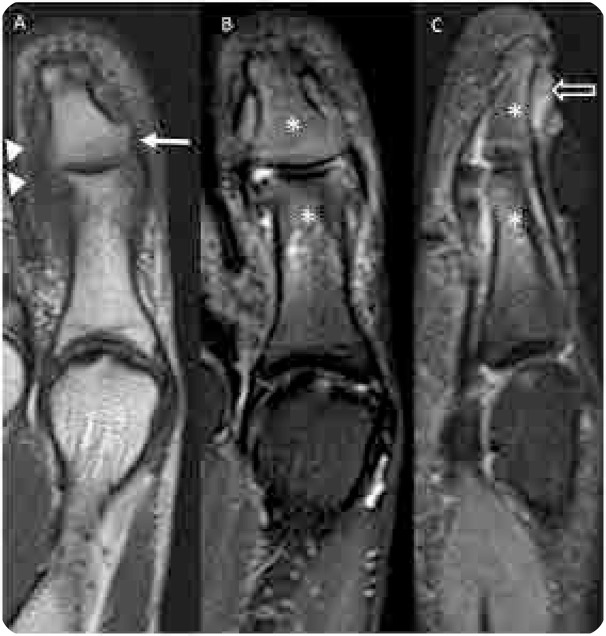
**(right).** Magnetic resonance imaging of the hallux. (**A**) Coronal T1-weighted image showing periarticular erosions (arrow) and soft-tissue swelling with intermediate to low signal intensity (arrowhead). Coronal (**B**) and sagittal (**C**) fat-suppressed proton density-weighted images depicting extensive bone-marrow oedema in the proximal and distal phalanges of the hallux (asterisks) related to the extensor and flexor enthesis, and oedema and thickening of the nail bed (open arrow in **C**).

A 42-year-old woman presented with hallux arthritis, nail dystrophy, edema, and erythema of the interphalangeal joint for 5 months after wearing tight shoes (**Figure 1**). She had recently received topical and oral antibiotics and anti-inflammatory treatments, which resulted in no clinical improvement. Her C-reactive protein level and erythrocyte sedimentation rate levels were 350 mg/L and 50 mm/h, respectively. On conventional radiography, periarticular erosions and periosteal reaction of the distal phalanx were identified (**Figure 2**). US revealed enthesitis characterised by hyperostosis of the proximal and distal phalanges, and increased thickness and hypoechogenicity of the extensor hallucis longus associated with peritendinous positive Doppler findings adjacent to the tendon’s insertion at the base of the distal phalanx. Synovitis of the interphalangeal joint of the hallux was also observed (Figure 3). On MRI of the foot, fat-suppressed proton density (FSPD)-weighted images depicted extensive bone-marrow oedema in the proximal and distal phalanges of the hallux related to the extensor and flexor enthesis, as well as oedema and thickening of the nail bed. Periarticular erosions and soft-tissue swelling with intermediate to low signal intensity on T1-weighted and FSPD images were also observed (**Figure 4**). Written informed consent was obtained from the patient.

## DISCUSSION

Psoriatic arthritis has been defined as inflammatory arthritis associated with psoriasis. Psoriatic onycho-pachydermo periostitis (POPP) is a rare subtype of psoriatic arthropathy that usually occurs in the small joints of the digits in association with cutaneous manifestations, onychodystrophy, painful soft-tissue thickening around the phalanx, and periosteal reaction.^1–6^

The diagnosis of POPP is based on clinical, laboratory, and imaging evaluations. POPP should be considered when periarticular erosions, periosteal reaction, new bone formation on the distal phalanges (e.g., a bony protuberance), and periostitis are observed on radiographs together with a history of arthritis, nail dystrophy, soft-tissue swelling, and erythema, as in our case. Soft-tissue thickening (dactylitis) and calcifications at tendon and ligament insertions (enthesopathy) might also be seen on radiographs.^2,5–9^

US can be used in the setting of POPP, as it might demonstrate inflammatory lesions of the disease. Bone changes such as hyperostosis can be visualised in association with tendon thickening, tenosynovitis and peritendinous positive Doppler findings. Moreover, US of the nail bed might demonstrate oedema and thickening.^2,10^

Recent advances in MRI have favoured an earlier and more accurate diagnosis of POPP, as this method can provide additional information in the evaluation of the disease. Extensive bone-marrow oedema, usually observed in the distal phalanx of the affected digit, can be demonstrated by intermediate to low signal intensity on T1-weighted images and high signal intensity on T2-weighted images. Periostitis is present when low-intensity periosteal thickening is observed on T1-weighted images, and soft-tissue swelling is present when intermediate to low signal intensity is seen on T1-weighted images and on fluid-sensitive sequences, with no associated oedema. Oedema and thickening of the nail bed might also be seen. The use of a contrast agent is not mandatory, but it can aid the assessment of POPP and its main differential diagnosis, which is infection.^2,4,5,6.11^

In the setting of monoarticular pain associated with erythema, and soft-tissue swelling, osteomyelitis should be considered. MRI has high sensitivity and specificity for the diagnosis of osteomyelitis, which cannot be made based on by physical examination and/or laboratory studies alone. Lesions of the early stage of the disease, such as bone-marrow oedema and Brodie’s abscess, as well as those related to the chronic stage, such as bone destruction and sequestrum of a necrotic fragment can be easily demonstrated on MRI. Additionally, complications involving tendons, ligaments and adjacent soft-tissue might also be seen. Contrast administration may be useful for the identification of intraosseous and soft-tissue abscesses.^2^

Periosteal thickening and extensive bone-marrow oedema originating from the enthesis without joint space involvement are important features of POPP that could aid its differentiation from osteomyelitis. Moreover, the soft-tissue swelling visualised in POPP presents as low signal intensity on both T1-weighted and fluid-sensitive sequences, with no associated edema.^2,4^

Although POPP has been considered to be a form of psoriatic arthritis, its pathogenesis remains poorly understood. It has been suggested that inflammation is transmitted from the involved articular cartilage to the insertions of ligaments and tendons. The anatomic relation of the extensor tendon, which fuses with the terminal phalanx with some of its fibers interdigitating the nail bed, may spread inflammation between the subungual dermis and the bone.^4,10^

Treatment for POPP has not yet been standardised. Most of the cases are treated with disease modifying anti-rheumatic drugs used for psoriatic arthritis, but this approach yields unsatisfactory outcomes in the majority of patients. POPP may also be treated with methotrexate and anti-tumour necrosis factor (TNF) agents. It was recently suggested that IL-17 inhibition could be an alternative biological therapeutic option, effective as TNF inhibition.^6,12^ Despite the clinical and imaging findings, our patient was treated with anti‐fungal medications for a long time at another facility, as reported for several other POPP cases in the literature.^3,10,13^ In our case, treatment with an anti-TNF agent was initiated, and a re-evaluation 4 weeks later revealed decreased pain scores.

In conclusion, MRI can depict POPP lesions very early in the course of the disease, with features such as extensive bone-marrow oedema and nail bed oedema and thickening, as seen in our patient. US and MRI have increased accuracy in diagnosing this condition earlier and have aid its differentiation from other diseases, such as osteomyelitis. The identification of this condition and initiation of appropriate treatment are crucial.^2,4,5^

## CONFLICT OF INTEREST

The authors declare no conflict of interest.

## AUTHOR CONTRIBUTIONS

Concept and design of the article: AS, FC

Data collection: LF, AS

Manuscript preparation: AS, CC

Critical revision for important intellectual content and approval of the version to be published: AS, EM, FC, CC

All co-authors take full responsibility for the integrity and accuracy of all aspects of the work.

## FINANCIAL SUPPORT

None.
